# Predicting Coral Species Richness: The Effect of Input Variables, Diversity and Scale

**DOI:** 10.1371/journal.pone.0083965

**Published:** 2014-01-15

**Authors:** Zoe T. Richards, Jean-Paul A. Hobbs

**Affiliations:** 1 Department of Aquatic Zoology, Western Australian Museum, Welshpool, Western Australia, Australia; 2 The Oceans Institute and School of Plant Biology, University of Western Australia, Crawley, Western Australia, Australia; New England Aquarium, United States of America

## Abstract

Coral reefs are facing a biodiversity crisis due to increasing human impacts, consequently, one third of reef-building corals have an elevated risk of extinction. Logistic challenges prevent broad-scale species-level monitoring of hard corals; hence it has become critical that effective proxy indicators of species richness are established. This study tests how accurately three potential proxy indicators (generic richness on belt transects, generic richness on point-intercept transects and percent live hard coral cover on point-intercept transects) predict coral species richness at three different locations and two analytical scales. Generic richness (measured on a belt transect) was found to be the most effective predictor variable, with significant positive linear relationships across locations and scales. Percent live hard coral cover consistently performed poorly as an indicator of coral species richness. This study advances the practical framework for optimizing coral reef monitoring programs and empirically demonstrates that generic richness offers an effective way to predict coral species richness with a moderate level of precision. While the accuracy of species richness estimates will decrease in communities dominated by species-rich genera (e.g. *Acropora*), generic richness provides a useful measure of phylogenetic diversity and incorporating this metric into monitoring programs will increase the likelihood that changes in coral species diversity can be detected.

## Introduction

A critical challenge facing conservation scientists and ecosystem managers is curtailing the loss of biodiversity in the face of rapid global change [1], [2]. Coral reefs support more than 35% of all known marine biodiversity, hence there is strong impetus to forecast, detect and mitigate losses in this ecosystem [3]–[5]. Coral reef biodiversity is however, at risk due to the recent destruction of 20% of the world's coral reefs and a further 50% of reefs in decline [6]. Consequently, there has been a dramatic increase in the threatened status of reef-building corals, with 33% of species now listed in elevated categories of threat by the IUCN [7]. The urgent need to conserve corals is emphasized further by the thousands of other marine species that rely on these habitat-forming organisms and the millions of people dependent on reefs for food security [8].

The task of protecting coral biodiversity is immense and exacerbated not only by the logistic challenges of conducting species-level surveys on SCUBA and the high level of taxonomic expertise needed to identify corals; but by the fact that coral communities are characterized by a large proportion of rare species [9]. Recent studies have shown that rare species disproportionately increase the potential breadth of functions provided by ecosystems across spatial scales [10], [11]. Thus, protecting rare species, and moreover, the full complement of species richness insures against future uncertainty arising from environmental change [11]. Despite species richness influencing ecosystem functioning, resilience and resistance to environmental change [12], for most coral communities, there is a critical shortage of rigorous species-level baseline data and that presents a major challenge for the conservation of diversity [13]–[16].

In the absence of species-level data, conservation decisions relevant to protecting coral biodiversity are based on subsets of data relating to indicator species [17], cross-taxon surrogates [18] or broad habitat-based proxy metrics [19]. Developing indicator, surrogate or proxy metrics that accurately represent trends in biodiversity is an important and pragmatic conservation objective [20]. While proxies reduce the time and cost required for data collection [21], [22], their effectiveness varies considerably and all have limitations [23], [24]. Hence, numerous studies have questioned the ability for proxy metrics to effectively represent biodiversity [18], [20]–[25] especially if their performance is not evaluated with empirical data [26].

On coral reefs, ‘reefscape proxies’ are commonly used to quantify the condition of coral reef habitat, with percent live hard coral cover being the most widely used metric in monitoring studies [27]–[29]. Despite its popularity, hard coral cover is not a robust indicator of coral biodiversity [30]. Subsequently, protecting reefs with a high level of coral cover may not have the expected flow-on biodiversity benefits. As such, there is a need to optimize the data collected in coral reef monitoring programs and find an effective way to make better predictions about the status of coral biodiversity.

In terrestrial systems, a high level of congruence has been documented between species and higher-taxon richness [31]–[38]. A similar significant positive linear relationship has been proposed to exist between species and generic richness in coral communities [30], [39]. Before generic richness can be broadly applied as a proxy indicator of species richness in coral communities, it is necessary for the relationship between these variables to be further characterized. To build on the initial findings of a companion study [30], here we examine if the linear relationship between generic and species richness is scale-dependent and how it is impacted by diversity.

The overall objective of this study was to examine the relationship between coral species richness and three potential proxy indicators: generic richness (measured on belt transects); generic richness (measured on point-intercept transects); and percent live hard coral cover (measured on point-intercept transects). Specifically, this study aims to: 1. Determine which proxy is the best predictor of species richness; 2. Determine if the relationships between predictor variables and species richness are affected by the spatial scale in which the data is analyzed and; 3. Determine whether the relationships between predictor variables and coral species richness vary between locations of varying diversity.

## Materials and Methods

### Ethics Statement

All necessary permits were obtained for the described field studies. Permits were obtained from the Department of Environment, Water and Heritage, Australia; the Marshall Island Marine Resource Authority; and the Kosrae Conservation and Safety Organization.

### Study Sites

From 2009 to 2012 underwater visual surveys of hermatypic scleractinian (hard) corals and percent coral cover were conducted at 14 sites at Majuro Atoll in the Republic of the Marshall Islands, Pacific Ocean (7°4′N 171°16′E); 22 sites at Kosrae, Micronesia, Pacific Ocean (5°19′N 162°59′E); and at eight sites within the Ashmore and Cartier National Marine Reserves, Timor Sea, NW Australia (Ashmore: 12°17′S 123°02′E; Cartier:12°32′S 123°33′E).

### Generic and Species Richness

In this study we examine observed species richness (the total number of species observed in a sample i.e. alpha-diversity) rather than estimated species richness which may be obtained through the application of statistical methods to correct for undetected species (e.g. Chao and jackknife estimators). Generic and species richness of reef building hard corals was documented on six replicate 50 m long ×2 m wide belt transects (i.e. 100 m per transect, 600 m per site). Belt transects were surveyed at two depths (three replicates at 3–5 m depth and three at 8–10 m depth). Within the belt transects, every coral colony (over 5 cm diameter) was identified to species level. Colonies under 5 cm were considered juveniles and not able to be identified in-situ because of the lack of skeletal development. In the case of large stands of coral (e.g. branching *Acropora*, large colonies of *Porites*), every 1 m^2^ was counted as a separate colony.

### Hard Coral Cover

The percent cover of hard corals was documented to generic level on point-intercept transects (PIT). This methodology involved resurveying the same transect used to document species richness (thus referred to here as ‘paired transects’). On the second pass of the transect tape, all benthos occurring directly below 100 uniformly distributed points (50 cm apart) per were recorded (see [30] for an illustration of the survey method).

### Data Analysis

The relationship between species richness (response variable) and 3 predictor variables {generic richness from belt transects (GRb), generic richness from point-intercept transects (GRp), and percent live hard coral cover (HCC)} were examined at two scales, site (mean across transects) and transect (total of all transects). Linear regression analyses were performed using the R statistical program version 2.15.3 [40]. The coefficient of determination (*R^2^*) was used as the measure of fit and standard errors indicate the accuracy of the prediction.

For strong (*R^2^*>0.7) and significant (p<0.0005) relationships, the regression equation is reported (*ŷ* = *a*+*bx*) (where *ŷ* represents mean species richness and *x* represents the predictor variable, *a* = intercept coefficient (±SE) and *b* represents *x* variable coefficient (±SE). Application of this equation enables species richness to be estimated. A series of one-way analyses-of-variance (ANOVA) were conducted on untransformed data to test the null hypothesis that there is no significant difference in the performance of predictor variables across scales of analysis (site versus transect).

## Results

A total of 193 ‘paired’ replicate transects were conducted at 3 locations (Table 1). In total, 19,300 m^2^ of coral reef habitat was surveyed and 29,406 colonies were identified. The highest species richness was at Ashmore Reef (191 species), followed by Kosrae (154 species) and Majuro (135 species). There was no significant relationship between species richness and the area surveyed (F = 121.06, df = 1, *p* = 0.058).

**Table 1:** The study locations, total number of colonies, total species and generic richness, total number of sites surveyed, transects per site and total number of paired transects per location.

Location	Number of colonies	Species richness	Generic richness	Number of sites	Number of transects per site	Total number of paired transects
Kosrae	10884	154	49	22	3	66
Majuro Atoll	9125	135	44	14	6 at 12 sites plus 3 at 1 site and 4 at 1site	79
Ashmore and Cartier Reefs	9397	191	51	8	6	48

The composition of the coral communities differed between the three locations. Ashmore Reef was dominated by colonies of *Acropora*, *Seriatopora*, *Porites* and *Montipora*; whilst Majuro was dominated by *Acropora*, *Porites* and *Pocillopora*, and Kosrae was dominated by *Porites*, *Acropora* and *Galaxea* (Figure S1a). Notable differences in the composition of communities at the three locations include the high abundance of *Porites* and *Galaxea* colonies at Kosrae; and there being twice as many *Montipora* spp. at Ashmore Reef than at the other locations. While *Acropora* colonies were commonly encountered at all three locations, there was far greater species-level diversity at Ashmore Reef and Majuro Atoll than observed at Kosrae (Figure S1b).

**Figure 1:**
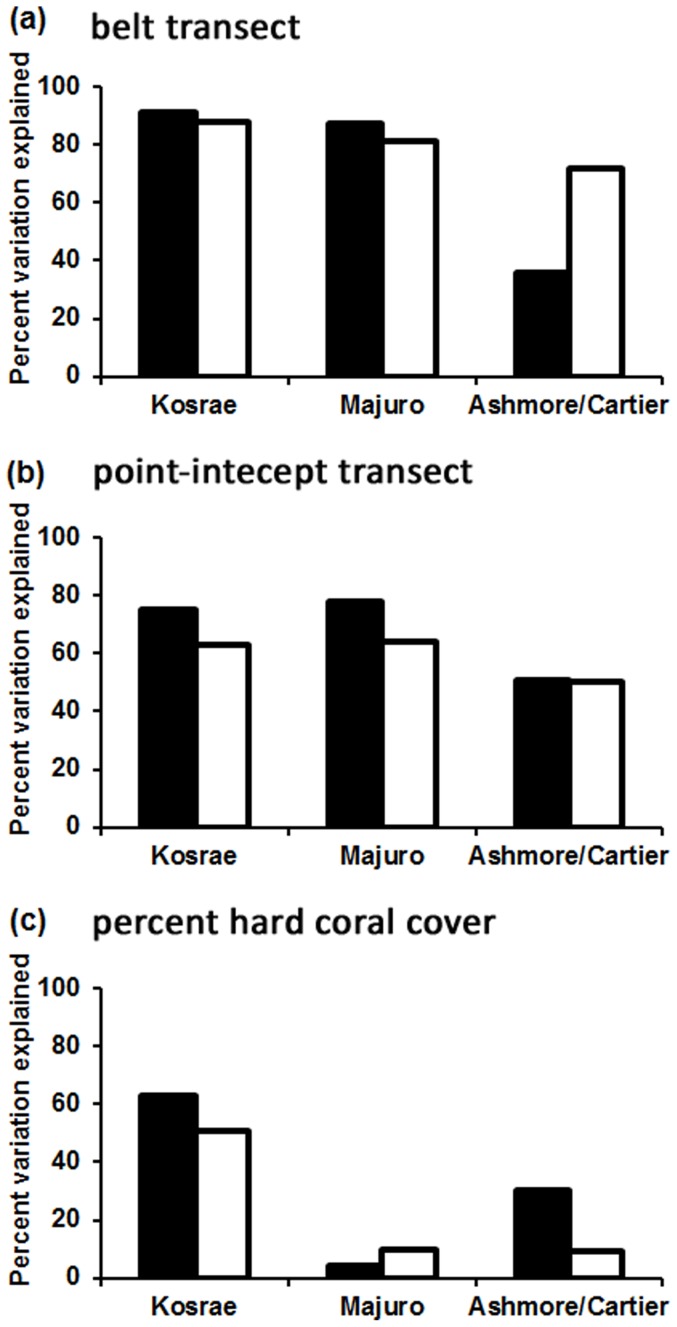
Histogram depicting the percent of variation in species richness that is explained by 3 predictor variables at 2 scales (black bars: site means; white bars: transect). (a) generic richness measured on a belt transect, (b) generic richness measured on a point-intercept transect, (c) percent live hard coral cover measured on a point-intercept transect.

**Figure 2:**
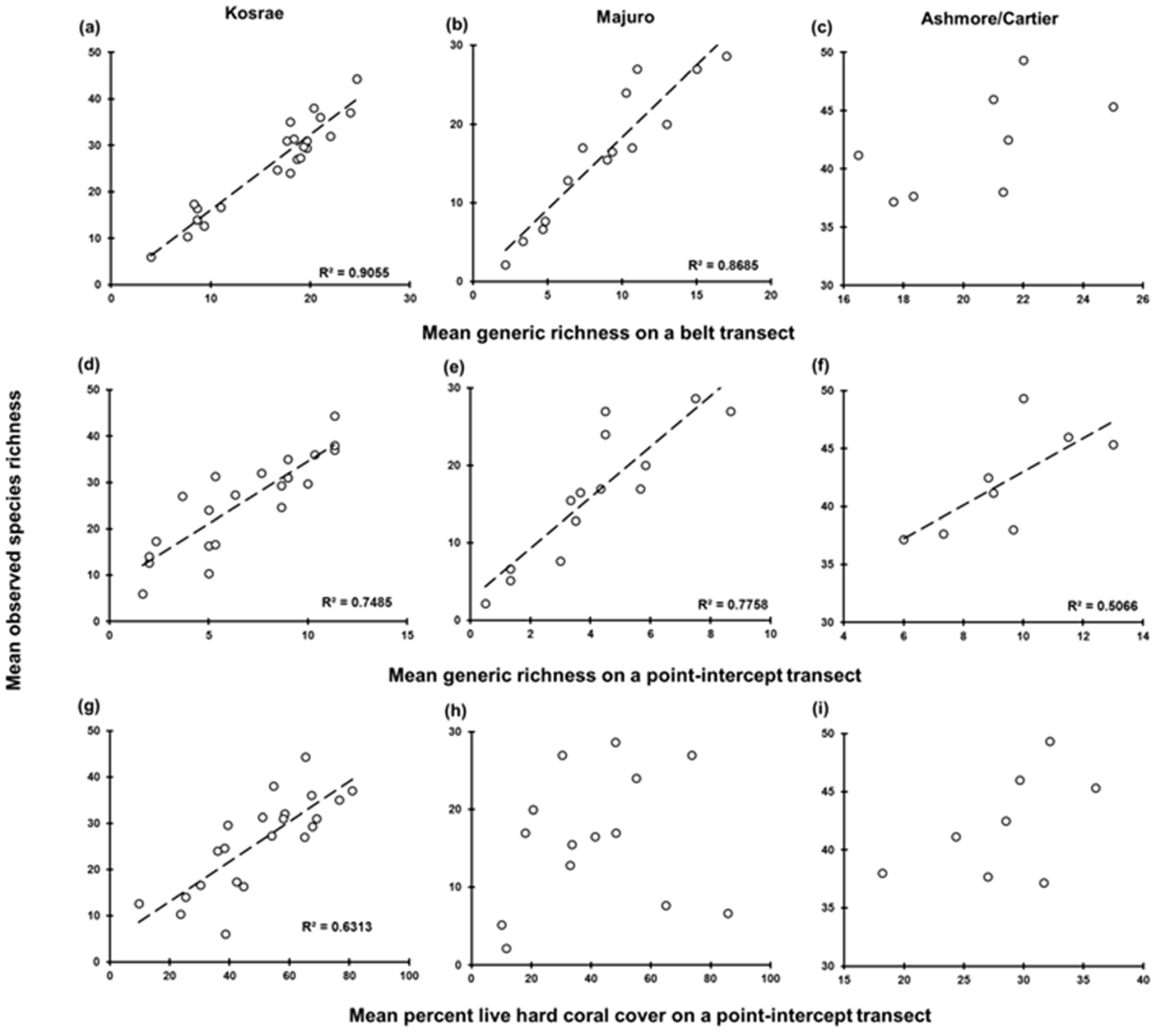
Site-level linear regressions of species richness on predictor variables (in rows) at the three locations (in columns). The number of sites for each location is shown in Table 1. *R^2^* and the 1∶1 relationship are shown for significant associations.

**Figure 3:**
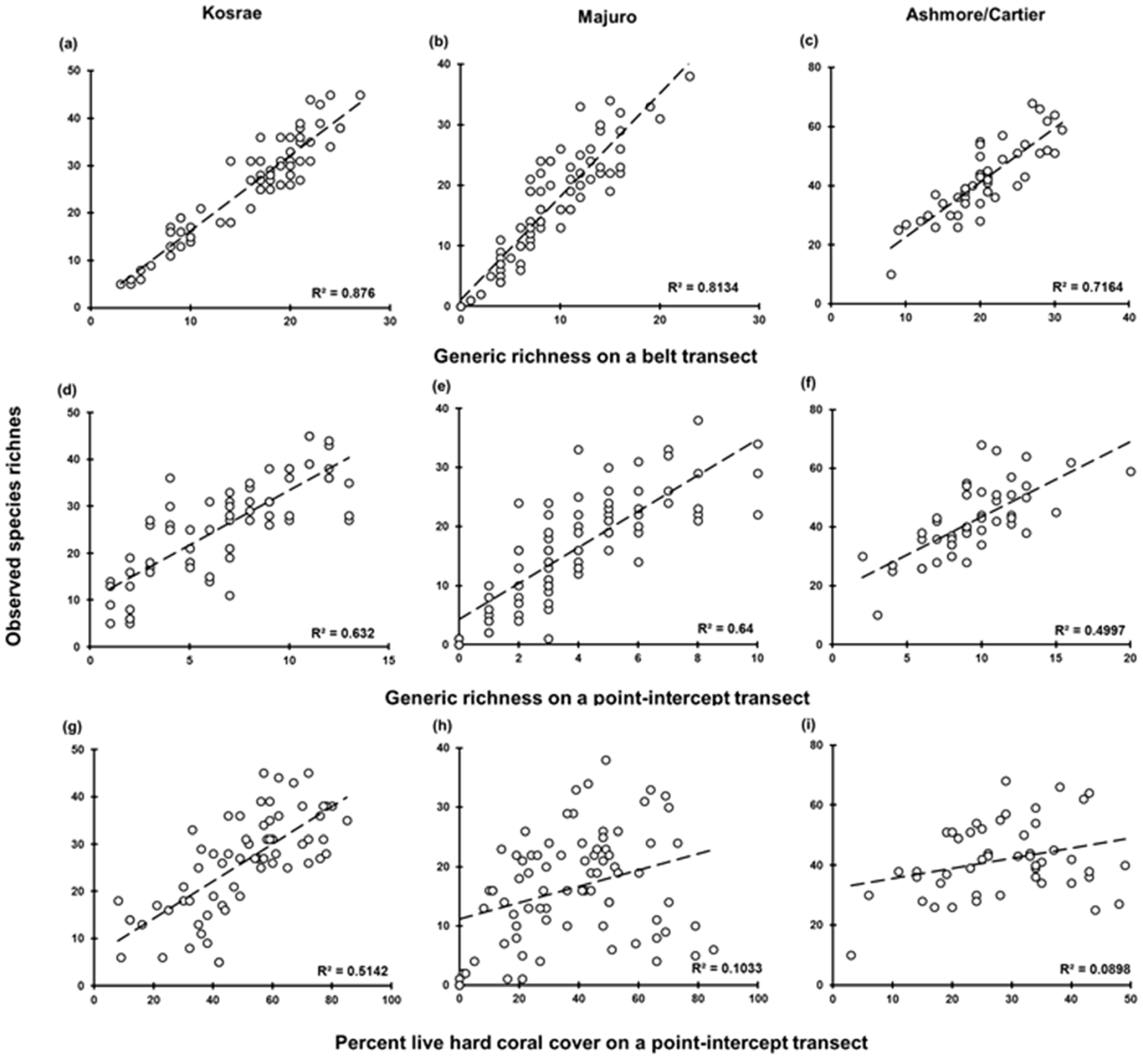
Transect-level linear regressions of species richness on predictor variables (in rows) at the three locations (in columns). The number of sites for each location is shown in Table 1. *R^2^* and the 1∶1 relationship are shown for significant associations.

The ability for input variables (HCC, GRp, GRb) to predict species richness varied considerably, ranging from 4–91% variation explained depending on location and scale examined. Overall, GRb provided the most accurate predictions of species richness as evidenced by the high R^2^ values (i.e. >70% variation explained in 5/6 analyses, Figure 1a; Figure 2a–c; Figure 3a–c), and highly significant linear relationships (p<0.001; Table 2). GRb is a particularly strong explanatory variable species richness at Kosrae where 91% of variation was explained based on site data (Table 2). Based upon this result, the regression equation from Kosrae site data is *ŷ* = 1.624 (±0.117)*x*−0.234 (±2.011) and from this equation species richness can be predicted (see Table 3).

**Table 2:** Regression statistics showing the strength and significance of linear relationships between species richness and a) Generic richness measured on a belt transect; b) Generic richness measured on a point intercept transect; and c) Percent live hard coral cover.

Location	Predictor Variable	Scale of Analysis	*R^2^*	*Adj R^2^*	SE	df	*F*-value	*P*-value	Sig.
KOSRAE	GRb	Site	0.905	0.901	3.189	21	191.576	1.050E-11	***
KOSRAE	GRb	Transect	0.876	0.874	3.718	65	452.115	1.035E-30	***
KOSRAE	GRp	Site	0.749	0.736	5.202	21	59.532	2.029E-07	***
KOSRAE	GRp	Transect	0.632	0.626	6.404	65	109.917	1.584E-15	***
KOSRAE	HCC	Site	0.631	0.613	6.299	21	34.251	1.003E-05	***
KOSRAE	HCC	Transect	0.514	0.507	7.358	65	67.750	1.263E-11	***
MAJURO	GRb	Site	0.868	0.858	3.243	13	79.240	1.240E-06	***
MAJURO	GRb	Transect	0.813	0.811	3.999	78	335.585	8.580E-30	***
MAJURO	GRp	Site	0.776	0.757	4.234	13	41.522	3.189E-05	***
MAJURO	GRp	Transect	0.636	0.631	5.588	78	134.299	1.493E-18	***
MAJURO	HCC	Site	0.038	−0.043	8.772	13	0.470	5.061E-01	ns
MAJURO	HCC	Transect	0.103	0.092	8.766	78	8.873	3.869E-03	**
ASHMORE	GRb	Site	0.360	0.253	3.863	7	3.371	1.160E-01	ns
ASHMORE	GRb	Transect	0.716	0.710	6.502	47	116.174	3.556E-14	***
ASHMORE	GRp	Site	0.507	0.424	3.391	7	6.161	4.767E-02	*
ASHMORE	GRp	Transect	0.500	0.489	8.635	47	45.953	1.957E-08	***
ASHMORE	HCC	Site	0.296	0.178	4.052	7	2.521	1.634E-01	ns
ASHMORE	HCC	Transect	0.090	0.070	11.647	47	4.538	3.853E-02	*

= 0.05;

= 0.005;

= 0.0005.

Strong (>0.70) and highly significant (p<0.0005) linear relationships are shaded.

**Table 3:** Regression equations for predicting species richness (*y*) based on generic richness (GR) on belt transects where *R^2^*>0.7.

	Regression equation	Range in *y* for *x* = 20	Range in *y* for *x* = 30	Range in *y* for *x* = 40
**Kosrae**				
Site data	*ŷ* = 1.624 (±0.117)*x*−0.234 (±2.011)	28–33	43–54	58–71
Transect data	*y* = 1.590 (±0.748)*x*+0.319 (±1.290)	16–48	24–72	33–95
**Majuro Atoll**				
Site data	*ŷ* = 1.829 (±0.206)*x*+0.033 (±2.015)	30–43	47–63	63–83
Transect data	*y* = 1.706 (±0.093)*x*+1.038 (±0.951)	32–38	48–56	65–74
**Ashmore-Cartier**				
Site data	*R^2^* too low to formulate a reliable equation			
Transect data	*y* = 1.846 (±0.171)*x*+4.171 (±3.620)	34–48	51–68	68–88

The scale upon which generic richness data was collected impacted upon the ability for species richness to be predicted. When data were collected on a point-intercept transect, generic richness provided modest to strong (*R^2^* = 0.50–0.78) and significant (*p*<0.05) positive linear relationships with coral species richness across all locations (Figure 2d–f; Figure 3d–f, Table 2). GRp provided a strong explanatory variable for species richness at Majuro Atoll with 78% of variation explained as a positive linear function based on the site data. At all other locations and scales of analysis however, R^2^ values were considered too low (i.e. below 0.7) and/or SE values were too high to provide meaningful or precise estimates of species richness.

Percent live hard coral cover was the least reliable predictor of coral species richness (4–63% variation explained, Figure 1c, Table 2). For HCC, both the strength and significance of the relationship with coral species richness varied. At Kosrae, a significant (p<0.001) linear relationship exists between HCC and coral species richness (*R^2^* = 0.51–0.63); while at Majuro and Ashmore/Cartier, the relationship between HCC and coral species richness was weak (*R^2^*<0.30) and of low, or no statistical significance (Figure 2g–i, Figure 3g–i, Table 2). Overall R^2^ values were considered too low (i.e. below 0.7) to provide meaningful estimates of coral species richness.

There was no significant difference (p<0.001) in the performance of GRb, GRp or HCC across the two scales of measurement (Table S1). Hence, analyzing the data at a finer spatial scale (i.e. transect level) did not necessarily increase predictive ability. There is one important exception however, at Ashmore Reef, the location with the highest number of species (see Table 1) the linear regression provided a better fit when analyzed at the transect level (*R^2^* = 0.72 for transect versus 0.36 for site, Figure 1, Table 2).

## Discussion

Evidence is mounting that there is limited redundancy in complex marine ecosystems like coral reefs [41]–[43]; and individual species can be surprisingly important to ecosystem resilience [44], [45]. Thus, even small changes in species diversity can have significant impacts on ecosystem function [46], [47]. Protecting species-level diversity is therefore a key component of maintaining the functional diversity of coral reefs. However, given that the task of species-level monitoring and management is immense, coral reef management authorities necessarily adopt ecosystem-based monitoring approaches. If managers of sensitive coral reefs are only informed at this level, the future of coral biodiversity could be jeopardized. Thus, striking a balance between ecosystem and species-level monitoring is an acute challenge to conservation science. This study confronts this challenge by examining the potential for 3 proxy metrics to represent species diversity.

It is standard practice for the status of coral reefs to be assessed based upon the level of hard coral cover [6], [27], [29], [48]–[51]. Under this model, a reef with high HCC is considered ‘healthy’ and a reef with low HCC is not. This study has confirmed however, that HCC is a poor linear predictor of coral species richness, thus a reef with high coral cover does not necessarily have high coral species diversity. Moreover, in apparent accordance with predictions of the intermediate disturbance hypothesis [52] at the locations examined here (see also [30]), species richness tends to peak at intermediate levels of coral cover rather than having a positive linear trend. Thus, the relationship between HCC and species richness is complex and this precludes its usefulness as a proxy indicator. Nevertheless, HCC remains an important habitat monitoring target because it has been correlated with other variables such as coral disease prevalence [53], and the abundance and composition of reef fishes [54] and overall reef condition [6], [27].

This dataset suggests that generic richness provides a more reliable indicator of species richness based upon the strong and significant linear relationships between these two variables. Since Veron [39] highlighted the association between generic and species richness in coral communities, there have been few empirical examinations of the nature of the relationship (but see [30]). Our study confirms that GRb provides a strong explanatory variable for the observed patterns of species richness at three Indo-Pacific locations of varying diversity. Thus, we conclude that GRb provides not only a tangible measure of phylogenetic diversity (which is in itself important for insuring against losses of evolutionary history [55], [56]); but a reliable proxy indicator of species richness.

In other taxa, the strength of the relationship between species and generic richness is influenced by scale, location; ecosystem complexity and community age [57]–[60]. Similarly, our results suggest the relationship between coral species and generic richness is sensitive to scale, most notably at locations where there is a high level of within-genus diversity. For example, at Kosrae and Majuro Atoll, GRb explained 81%–91% of the variation in species richness regardless of the scale of analysis. Whereas at Ashmore Reef GRb performed less reliably, explaining only 36% of the variation at a site level (and 72% at a transect level). The variable performance of GRb at Ashmore Reef in comparison to the other localities relates to the differences in diversity and community structure at this location. Ashmore Reef has the highest level of species richness among the locations examined, and the community is dominated by species from diverse genera such as *Acropora* and *Montipora* (Figure S1). Conversely, the Kosrae and Majuro communities are dominated by less diverse genera including *Porites* and *Pocillopora*. Therefore, if species-rich genera dominate the community, the strength of the generic-species richness relationship diminishes.

Moreover, for a community comprised of mono-specific genera there is an exact relationship between generic and species richness. As the number of species in each genus increases, the strength of the relationship with species richness decreases. Therefore, a limitation of using generic richness as a proxy indicator of species richness is that the accuracy of estimates will decrease in communities dominated by species-rich genera. Conducting the regression analysis at a finer spatial scale (e.g. transect level) can help improve the predictive capability of GRb; however the equal weighting of genera regardless of number of species within, is an important limitation of the GRb proxy approach which detracts from its usefulness, especially in diverse tropical communities that are dominated by species-rich genera.

Considering species-level data collection is not feasible for corals within the scope of most coral reef monitoring programs, we advocate that generic richness data should be collected for numerous reasons. Firstly, it provides a measure of phylogenetic diversity. Secondly, if applied with caution, GRb data can be used to obtain a robust estimate of species richness; and thirdly, generic richness is easily quantified. Although it takes longer to document generic richness on a belt transect than to collect generic richness or benthic cover data on a point-intercept transect, it is possible for a suitably trained diver to complete up to six 50×2 m belt transects per 60 minute dive. While identifying corals to genus is far easier than identifying to species, para-taxonomic training is still necessary because there are 86 genera of scleractinian corals in the Indo-Pacific. However, genus-level identification guides are available and it is realistic for field guides to be taken underwater to help confirm identifications.

Despite its potential as a proxy indicator, caution must be applied when using GRb data to predict species richness because it is still a relatively coarse measure and the level of precision obtained is contingent on numerous conditions. For example, at Kosrae, if a mean of 20 genera were recorded across a site, we would predict between 28–33 species were present. With application of the regression equation based upon the transect data, a far less precise estimate is obtained (16–48 species, see Table 3). Thus, even if the R^2^ is relatively high, at sites with high heterogeneity, the error about the coefficient of determination may be large; hence the range of predicted species richness may be so great that it becomes non-informative. Furthermore, if species-rich genera dominate the community, the strength of the generic-species richness relationship diminishes. Lastly, in the context of threatened species, generic richness data will not provide the necessary information to enable species population trends to be monitored.

## Conclusion

If logistic or budgetary constraints prohibit ongoing species-level monitoring of coral biodiversity, measuring generic richness on replicated belt transects provides a meaningful way to monitor and detect critical changes in phylogenetic diversity and to predict species richness with reasonable amount of certainty. It is important to note however, that prior to incorporating GRb into monitoring programs, species level data should first be collected to confirm that generic richness provides a robust and precise estimate of species richness at the target location. Furthermore, the most appropriate scale of analysis must be resolved and the performance of the proxy metric must be regularly evaluated, particularly after local disturbances. Overall, if preventing coral biodiversity loss is a priority to coral reef management authorities, it is essential that monitoring programs are adapted to include a suitable proxy for species richness that could complement broad-scale habitat data.

## Supporting Information

Figure S1 Composition of the coral communities at the three study locations. (a) Total number of colonies on belt transects within each genus; (b) Species richness within the 10 most species-rich genera.
(TIF)

Table S1 ANOVA for examining the performance of predictor variables across scales of analysis (site versus transect).
(DOCX)
