# Supplementary figures and images for: Predicting Coral Species Richness: The Effect of Input Variables, Diversity and Scale
Source: PLoS One. 2014 Jan 15;9(1):e83965. doi: 10.1371/journal.pone.0083965 (PMC3893078; doi:10.1371/journal.pone.0083965)

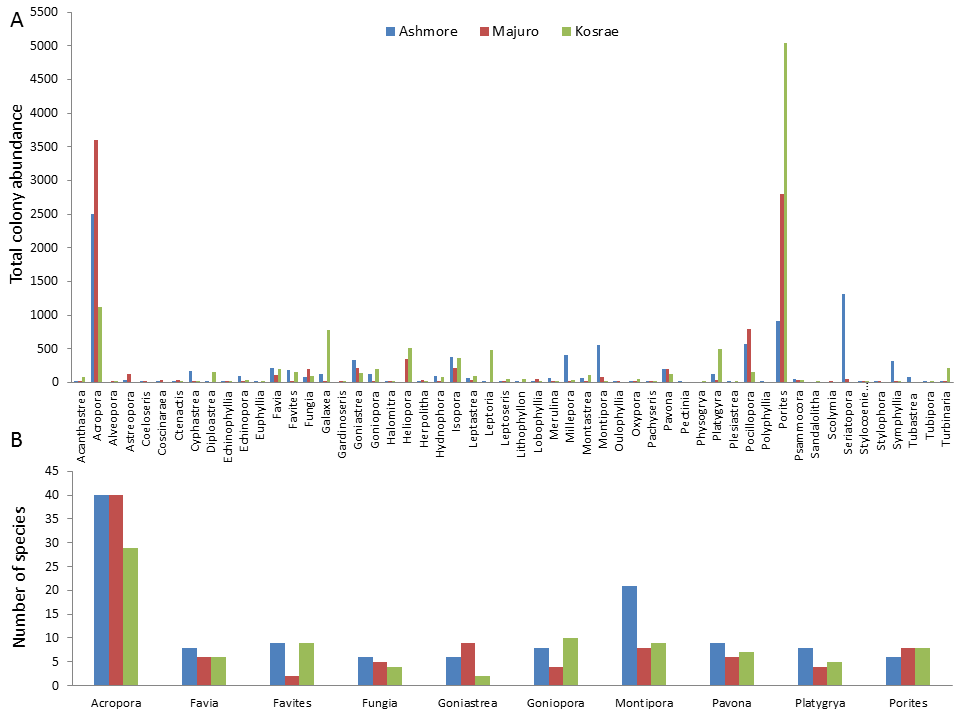

Supplement: Figure S1 — Composition of the coral communities at the three study locations. (a) Total number of colonies on belt transects within each genus; (b) Species richness within the 10 most species-rich genera. (TIF) [file pone.0083965.s001.tif]
