# Supplementary material for: Predicting Coral Species Richness: The Effect of Input Variables, Diversity and Scale
Source: PLoS One. 2014 Jan 15;9(1):e83965. doi: 10.1371/journal.pone.0083965 (PMC3893078; doi:10.1371/journal.pone.0083965)
Supplement: Table S1 — ANOVA for examining the performance of predictor variables across scales of analysis (site versus transect). (DOCX) [file pone.0083965.s002.docx]

| Predictor Variable | df | *F*-value | *P*-value |
| --- | --- | --- | --- |
| HCC | 1 | 3.77 | 0.303 |
| GRp | 1 | 233.01 | 0.042 |
| GRb | 1 | 7.05 | 0.217 |
